# 0866. Repercussion in hemodynamic values and extravascular lung water of alveolar recruitment maneuvers, in an experimental model of ARDS

**DOI:** 10.1186/2197-425X-2-S1-P66

**Published:** 2014-09-26

**Authors:** M Varela Durán

**Affiliations:** Surgery, Autonoma University, Madrid, Spain; Complejo Hospitalario Universitario de Pontevedra, Anesthesia and Critical Care, Pontevedra, Spain

## Objectives

To demonstrate the hemodynamic changes caused by different conducted maneuvers of alveolar recruitment, in 17 pigs with ARDS, provoked experimentally.

## Methods

Our Study was carried out in 17 Landrace pigs with ARDS, evoked through multiple bronchoalveolar lavages with saline serum. Three groups of study were established according to the maneuvers of alveolar recruitment (AR) made after the development of the ARDS.

### A group

"Total AR" realizing maneuvers of recruitment to obtain PaO2>90% of the basal value, with various maneuvers of "rapid AR" and one maneuver of "sustained insufflation".

### B group

simple maneuver of "rapid AR" without objetive of oxigenation.

### C group

Control group: without RA, using PEEP values bellow LIP (lower inflection point).

The hemodynamic study (Heart rate, mean BP, PCP, PAP, CI), EVLW, and lactic acid, was made at basal time and at 15, 60, 180 and 360 minutes, after the provocation of ARDS. The catheters used for hemodynamic monitoring consisted in: venous catheters, arterial catheters and pulmonary artery catheter for determination of cardiac output (Edwards Swan-Ganz thermodilution catheter). The hemodynamic monitoring equipment (Drager PM 8060 Vitara) and the PiCCO equipment was used for monitoring Cardiac output, circulatory and cardiopulmonary variables and EVLW (Extravascular Lung Water). Statistical analysis was made using ANOVA Study, T Student, X ^2^, and exact Test of Fisher.

## Results

In our Study, the observed hemodynamic values: HR, mean BP, PCP, PAP, CI, were similar using a "Total" or "Parcial" recruitment maneuver and also using a value of PEEP below the lower inflection point (LIP). The use of a PEEP value, below the LIP,without RA maneuvers, in experimental ARDS occurred after bronchoalveolar lavage, obtained differences in the values of mPAP. No significant differences in lactic acid in the three study groups were observed. The EVLW has a tendency to decrease with the "Total" or "Parcial" alveolar recruitment maneuvers.

## Conclusions

We recommended AR maneuvers associated with the use of a PEEP value below the LIP, in the ventilatory management of ARDS.

